# In Vitro Determination of the Main Effects in the Design of High-Flow Nasal Therapy Systems with Respect to Aerosol Performance

**DOI:** 10.1007/s41030-018-0054-x

**Published:** 2018-06-01

**Authors:** Gavin Bennett, Mary Joyce, Louise Sweeney, Ronan MacLoughlin

**Affiliations:** Aerogen Limited, IDA Business Park, Dangan Galway, Ireland

**Keywords:** Aerosol, Design of experiments, Droplet size, Emitted dose, Flow rate, High-flow nasal therapy, Nebulizer

## Abstract

**Introduction:**

The use of concurrent aerosol delivery during high-flow nasal therapy (HFNT) may be exploited to facilitate the delivery of a variety of prescribed medications for inhalation. Until now, a systematic approach to determine the conditions required to yield an optimal emitted dose has not been reported. The aim of this study was to establish the effects of gas flow rate, input droplet size, and nebulizer position on the amount of aerosol exiting the nasal cannula during HFNT and thus becoming available for inhalation.

**Methods:**

Testing was completed according to a factorial statistical design of experiments (DOE) approach. Emitted dose was characterized with a vibrating mesh nebulizer (Aerogen Solo, Aerogen Ltd) for an adult model of HFNT at three clinically relevant gas flow rates, using three nebulizers producing varying input droplet sizes and placed at two different nebulizer positions.

**Results:**

Increasing the gas flow rate significantly lowered the emitted dose, with a dose of 7.10% obtained at 10 LPM, 2.67% at 25 LPM, and 1.30% at 40 LPM (*p* < 0.0001). There was a significant difference in emitted dose between nebulizers with different input droplet sizes, with increasing input droplet size associated with a reduced emitted dose (6.11% with an input droplet size of 3.22 µm, 2.76% with 4.05 µm, and 2.38% with 4.88 µm, *p*  = 0.0002, Pearson’s *r* = − 0.2871). In addition, the droplet size exiting the nasal cannula interface was lower than that produced by the aerosol generator for all devices under test. Positioning the nebulizer immediately after the humidification chamber yielded a marginally greater emitted dose (3.79%) than when the nebulizer was placed immediately upstream of the nasal cannula (3.39%). Flow rate, input droplet size, and nebulizer position were at the 0.10 level of significance, indicating that all three factors had significant effects on emitted dose. According to the DOE model, flow rate had the greatest influence on emitted dose, followed by input droplet size and then nebulizer position.

**Conclusion:**

Our findings indicate that in order to optimize the amount of aerosol exiting the nasal cannula interface during HFNT, it is necessary for gas flow rate to be low and the input droplet size to be small, while the nebulizer should be positioned immediately after the humidification chamber.

**Funding:**

Aerogen Limited.

## Introduction

High-flow nasal therapy (HFNT) is a means of delivering heated humidified air/oxygen mixes to patient airways that allows for higher flows than conventional low-flow therapy [1]. HFNT provides sufficient flow rates to equal or exceed patient inspiratory flow and reduces the inspiratory resistance associated with the nasopharynx, thus reducing the work of breathing [2]. Humidification during HFNT is essential for proper function of the epithelial lining and is an accepted standard of care. Without humidification, unidirectional inspiratory nasal airflow may lead to the drying of mucosa and the release of inflammation mediators [3]. Delivering inhaled medications to the lungs from a nasal cannula interface is particularly advantageous for medications with extended delivery times or a frequent dosing regimen. The use of concurrent aerosol delivery during HFNT may be exploited to facilitate the delivery of a variety of prescribed medications for inhalation. These include but are not limited to β-agonists and hypertonic saline [4, 5]. The mounting observations of response and clinical evidence in combination with its ease of use and patient tolerability have resulted in increasing adoption of HFNT, with particular interest in concurrent aerosol delivery during HFNT [6–10]. Morgan et al. showed that infants with acute bronchiolitis tolerated aerosolized β-agonist therapy better during HFNT than with a facemask, possibly preventing them from escalating to more invasive respiratory support [11]. In vitro studies indicate that aerosols can be efficiently delivered during HFNT [12, 13]. However, several factors influence both the amount of aerosol exiting the cannula and the amount available to the patient. These factors include the rate of gas delivered, the size of the nasal prongs, humidification, the size of the aerosol droplets, and the aerosol generator type and position [12–15].

Bhashyam et al. showed that aerosols can be delivered from a mesh nebulizer (Aerogen Solo, Aerogen, Ireland) through infant, pediatric, and adult nasal cannula at an oxygen flow rate of 3 LPM. However, gas flow rates of 30–50 LPM are more commonly used for HFNT in adults [12]. Ari et al. reported aerosol delivery from a mesh nebulizer (Aerogen Solo) through an Optiflow (Fisher & Paykel, Auckland, New Zealand) pediatric nasal cannula at flow rates of 3–6 LPM with oxygen or heliox. The highest aerosol efficiency of ~ 10% was achieved at a flow rate of 3 LPM, and this decreased significantly to ~ 2% (with oxygen) as the flow rate was increased to 6 LPM [13]. Both of the aforementioned studies reported depositional losses in the delivery system in the range of 75–98% [12, 13]. Perry et al. reported the in vitro inspired dose of albuterol from a mesh nebulizer (Aerogen Solo) through a humidified HFNT system (Vapotherm, Stevensville, MD, USA). The inhaled doses (percent of the nominal dose) were 2.5%, 0.8%, 0.4%, and 0.2% for the adult cannula at 5, 10, 20, and 40 LPM, respectively. The authors concluded that high flow rates, small nasal prongs, humidification, and sharp changes in the direction of aerosol flow contributed to a low efficiency of in vitro aerosol delivery [15].

Several approaches to improve aerosol delivery during HFNT have been explored. During HFNT, much aerosol is lost due to impaction in the circuit due to the high flows used [14]. Longest et al. have attempted to generate submicron particles, which may reduce the chances of impaction in the circuit but it also reduces the drug mass available to the lungs [16, 17]. These studies notwithstanding, clinicians and users would benefit from a comprehensive review of the relevant factors that affect the amount of aerosol exiting the cannula. A systematic approach to establish the conditions required to yield an optimal emitted dose has not yet been reported. The objective of this study was to determine, using the well-established design of experiments approach, the main effects—those of the gas flow rate, input droplet size, and nebulizer position—on the amount of aerosol made available to the patient for inhalation during HFNT.

## Methods

### HFNT Circuit and Interfaces

**Fig. 1:**
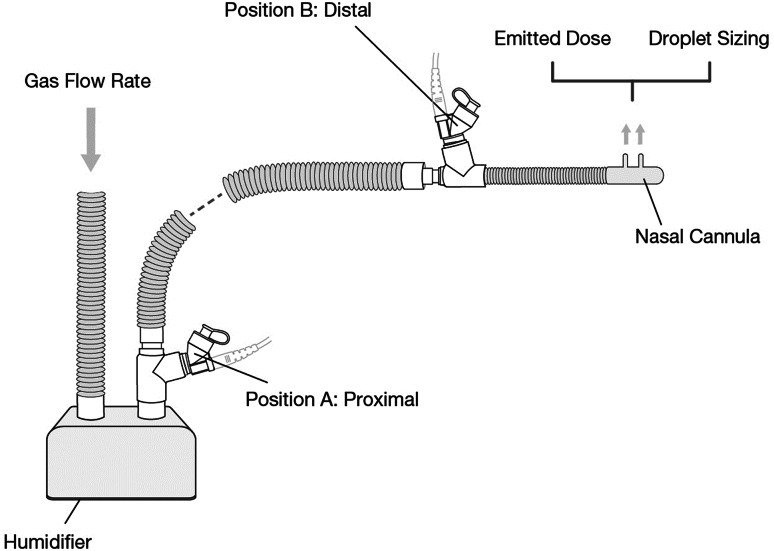
Illustration of test setup employed, indicating the two nebulizer placement positions within the HFNT circuit

The Hudson RCI Neptune system (Teleflex Medical, High Wycombe, UK) was used with adult (P/N: 870-19KIT, ID 22 mm, length 1524 mm), pediatric (P/N: 780-30, ID 15 mm, length 1524 mm), and infant (P/N: 780-01, ID 10 mm, length 1524 mm) heated wire circuits. Adult (P/N: 2411-01, ID 4 mm, length 500 mm), pediatric (P/N: 2411-02, ID 3 mm, length 400 mm), and infant (P/N: 2411-03, ID 2 mm, length 300 mm) nasal cannulas (Comfort Flo, Teleflex Medical) were used. Nebulizers were placed in the circuit using a standard T-piece connector positioned either proximally (immediately after the humidification chamber) or distally (immediately upstream of the cannula), as illustrated in Fig. 1.

### Nebulizers

Experiments were performed with three vibrating mesh nebulizers (Aerogen Solo). The nebulizer performance characteristics, measured using laser diffraction (Spraytec, Malvern Instruments, Malvern, UK) as previously described [18], are outlined in terms of average input droplet size (µm volumetric median diameter, VMD) and aerosol output rate (mL/min) (Table 1).

**Table 1:** Input droplet size and output rate data with 0.9% saline (*N* = 3), direct from the nebulizer

Input droplet size (µm VMD)	Aerosol output rate (mL/min)
3.22 ± 0.02	0.27 ± 0.02
4.05 ± 0.02	0.34 ± 0.03
4.88 ± 0.03	0.51 ± 0.02

### Design of Experiments

Testing was completed according to a factorial statistical design of experiments (DOE) approach (Minitab 17 statistical software, 2010). Experimental runs (*n* = 60 in total) were created to fit a quadratic model, in order to establish the conditions necessary to maximize the emitted dose with an adult nasal cannula. The DOE approach automatically randomized the order in which experimental runs were completed. Randomization balances the effects of uncontrollable conditions and reduces the chance that these conditions will bias the results. Thus, the sample number for each experimental run is dictated by the DOE. A summary of the DOE study design is shown in Table 2. Inputs used included gas flow rate, droplet size, and nebulizer position within the circuit.

**Table 2:** Study design obtained using a design of experiments approach

Base design	3 Factors, 8 runs
Replicates	4
Center points	20
Axial points	8
Total runs	60
Blocks	5

### Emitted Dose

Testing was performed in accordance with the DOE design presented in Table 3. Emitted dose was characterized during HFNT at three gas flow rates, using three nebulizers of varying input droplet size at two different nebulizer placement positions. Positions were chosen to reflect previous nebulizer placements in the literature. The nasal prongs from the cannula were placed distal to a filter (RespirGard II 303, Baxter, Ireland). To eliminate the potential for rained-out aerosol or humidity reaching the filter, a curved elbow was placed between the nasal prongs and the filter. This ensured that only aerosol droplets entrained in the gas flow were captured on the filter. The humidifier was powered on and allowed to come to temperature (37 °C) and a 2 mL dose of albuterol sulphate (2 mg/mL) was nebulized. Albuterol was used as it is a commonly nebulized formulation used in the characterization of aerosol drug delivery systems, and is specified for use as a tracer aerosol in the international standard ISO 27427:2013 [19]. At the end of each dose, the drug captured on a filter was eluted using 10 mL of deionized water. The mass of drug was quantified by means of UV spectrophotometry at a wavelength of 276 nm and interpolation on a standard curve of albuterol sulphate concentrations (100–3.125 µg/mL). Results for emitted dose were expressed as the percentage of the nominal dose placed in the nebulizer’s medication cup.

**Table 3:** Summary of testing completed to determine the effects of input droplet size, delivery gas flow rate, and nebulizer position on emitted dose using an adult cannula

Input droplet size (µm VMD)	Delivery gas flow rate (LPM)	Nebulizer position	Sample number (*n*)
3.22	10	Proximal	*n* = 4
		Distal	*n* = 4
	25	Proximal	*n* = 0
		Distal	*n* = 1
	40	Proximal	*n* = 4
		Distal	*n* = 4
4.05	10	Proximal	*n* = 1
		Distal	*n *= 1
	25	Proximal	*n *= 10
		Distal	*n *= 10
	40	Proximal	*n* = 1
		Distal	*n* = 1
4.88	10	Proximal	*n* = 4
		Distal	*n* = 4
	25	Proximal	*n* = 1
		Distal	*n* = 1
	40	Proximal	*n* = 4
		Distal	*n* = 4

## Emitted Doses at Pediatric and Infant Cannulas

In order to further investigate the effects of gas flow rate, input droplet size, and nebulizer position on emitted dose, testing was conducted using pediatric and infant nasal cannulas. The entire DOE was not completed for these cannulas—only the extremes in terms of flow rate, droplet size, and nebulizer position. Flow rates were adjusted in accordance with the manufacturer’s instructions. Testing was performed as outlined in Tables 4 and 5.

**Table 4:** Summary of testing completed to determine the effects of input droplet size, delivery gas flow rate, and nebulizer position on emitted dose using a pediatric cannula

Input droplet size (µm VMD)	Delivery gas flow rate (LPM)	Nebulizer position	Sample number (*n*)
3.22	5	Proximal	*n* = 3
		Distal	*n* = 3
	20	Proximal	*n* = 3
		Distal	*n *= 3
4.05	12.5	Proximal	*n* = 3
		Distal	*n* = 3
4.88	20	Proximal	*n* = 3
		Distal	*n* = 3

**Table 5:** Summary of testing completed to determine the effects of input droplet size, delivery gas flow rate, and nebulizer position on emitted dose using an infant cannula

Input droplet size (µm VMD)	Delivery gas flow rate (LPM)	Nebulizer position	Sample number (*n*)
3.22	2	Proximal	*n* = 3
		Distal	*n* = 3
	8	Proximal	*n* = 3
		Distal	*n* = 3
4.05	5	Proximal	*n* = 3
		Distal	*n *= 3
4.88	8	Proximal	*n *= 3
		Distal	*n* = 3

## Influence of Nasal Cannula Design

The emitted dose was compared between two adult nasal cannula interfaces (Comfort Flo; P/N: 2411-01 and Comfort Flo Plus; P/N: 2412-12, Hudson RCI Neptune, Teleflex Medical) when used in combination with the Hudson RCI Neptune circuit. The Comfort Flo cannula has an ID of 4 mm and length of 500 mm, while the Comfort Flo Plus cannula has an ID of 10 mm and length of 270 mm. A nebulizer of midpoint input droplet size (4.05 µm) was used. Testing was completed at three gas flow rates (10, 25, and 40 LPM). For the purposes of this test, the nebulizer was placed proximally within the HFNT circuit (wet side of the humidifier).

## Aerosol Droplet Size Measurement

In order to characterize the potential aerosol distribution available to the patient, the mass median aerodynamic diameter (MMAD) and geometric standard deviation (GSD) were characterized for selected test combinations (Next Generation Impactor, Copley Scientific, Nottingham, UK), as per Pharmacopeia 34 <601> [20]. The Aerogen Solo nebulizer and standard T-piece were presented to the throat of the impactor while operating at a vacuum of 15 LPM. A 2 mL dose of albuterol sulphate (2 mg/mL) was nebulized and drug was eluted from the collection stages of the impactor using 10 mL of deionized water.

The MMAD at the nasal cannula outlet, across two delivery gas flow rates, was characterized as previously reported [21] by presenting the cannula to the impactor throat while operating at a vacuum of 15 LPM. The cannula was held in place against the opening of the impactor throat in a repeatable manner using a custom fixture. The Aerogen Solo nebulizer was positioned proximally in the HFNT circuit. MMAD was characterized at delivery gas flow rates of 10 and 40 LPM (*n* = 3). MMAD and GSD were calculated using the validated CITDAS software (Copley Scientific, UK).

## Statistical Data Analysis

A one-way analysis of variance (ANOVA) was conducted to establish if the emitted dose varied significantly depending on the gas flow rate and when nebulizers with different input droplet sizes were used. A multiple comparison test (Tukey test) was performed to compare the mean of each column with the mean of every other column when comparing the doses emitted with different gas flow rates and by nebulizers of different input droplet sizes. A Student’s *t* test was performed to determine if there was a significant difference in emitted dose between nebulizer positions in the circuit. *p* values of < 0.05 were considered statistically significant.

When testing was completed according to the statistical DOE approach, a bar graph of standardized effects was generated. If factors in the quadratic model reached the 0.10 level of significance, it was concluded that there was a significant relationship between those factors and emitted dose. Factors with longer bars in the graph had a greater influence on emitted dose. The prediction and optimization report showed factor settings that optimized emitted dose as well as alternative solutions that were nearly optimal.

## Compliance with Ethics Guidelines

This article does not contain any studies with human participants or animals performed by any of the authors.

## Results

### Emitted Dose

The mean ± standard deviation values of emitted dose (%) from the adult nasal cannula are outlined in Table 6. The emitted dose was assessed during HFNT at three different flow rates (10, 25, and 40 LPM), using three nebulizers with different input droplet sizes (3.22, 4.05, and 4.88 µm) and at two different nebulizer placement positions (proximal and distal).

**Table 6:** Mean ± standard deviation values of emitted dose (%) for testing completed according to the DOE using an adult nasal cannula (Comfort Flo P/N: 2411-01, Teleflex Medical)

Input droplet size (µm VMD)	Flow rate (LPM)	Nebulizer position	Sample number (*n*)	Emitted dose (%)
3.22	10	Proximal	*n* = 4	11.63 ± 0.39
		Distal	*n* = 4	8.75 ± 0.69
	25	Proximal	*n* = 0	n/a
		Distal	*n* = 1	2.79
	40	Proximal	*n* = 4	2.68 ± 0.81
		Distal	*n* = 4	0.70 ± 0.34
4.05	10	Proximal	*n* = 1	6.62
		Distal	*n* = 1	4.04
	25	Proximal	*n* = 10	2.62 ± 0.77
		Distal	*n* = 10	2.61 ± 0.66
	40	Proximal	*n* = 1	0.77
		Distal	*n* = 1	1.25
4.88	10	Proximal	*n* = 4	2.75 ± 0.52
		Distal	*n* = 4	5.04 ± 0.52
	25	Proximal	*n* = 1	1.80
		Distal	*n *= 1	1.91
	40	Proximal	*n* = 4	1.11 ± 0.40
		Distal	*n* = 4	0.88 ± 0.19

**Fig. 2:**
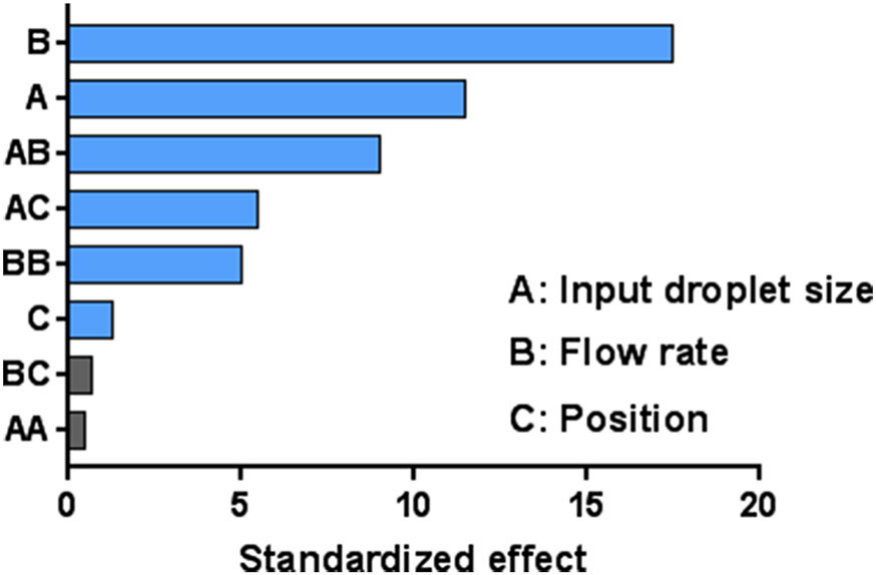
Effects of flow rate, input droplet size, and nebulizer position on emitted dose.* Blue bars* in the graph show the factors that had significant effects on the emitted dose (at the 0.1 level of significance).* Gray bars* represent nonsignificant terms that were removed from the model. Flow rate had the greatest influence on emitted dose, followed by input droplet size and then nebulizer position

A bar graph of standardized effects was generated by the DOE model (Fig. 2). Gas flow rate, input droplet size, and nebulizer position were at the 0.10 level of significance, indicating that all three factors had a significant effect on emitted dose. Based on the results presented, gas flow rate had the greatest effect on emitted dose, followed by input droplet size and then nebulizer position. The optimal settings to maximize the emitted dose were as follows: input droplet size of 3.22 µm, gas flow rate of 10 LPM, and a proximal nebulizer position at the humidifier. The graph also highlights the effects when the settings of two factors were changed together. Altering the settings of input droplet size and gas flow rate (AB) or of input droplet size and nebulizer position (AC) simultaneously significantly affected the amount of drug exiting the cannula outlet. The main aim of this study was to establish how changes to a single factor affected the mean emitted dose. The prediction and optimization report showed factor settings that optimized the emitted dose as well as alternative solutions that were nearly optimal, as outlined in Table 7. The model explained 92.80% of the variation in emitted dose.

**Table 7:** Top five alternative solutions to maximize emitted dose with an adult nasal cannula (Comfort Flo P/N: 2411-01, Teleflex Medical)

Input droplet size (µm VMD)	Flow rate (LPM)	Nebulizer position	Predicted emitted dose (%)
3.22	10	Distal	8.91
4.05	10	Proximal	7.07
4.05	10	Distal	6.64
3.22	25	Proximal	5.38
4.88	10	Distal	4.37

### Influence of Gas Flow Rate on Emitted Dose

**Fig. 3:**
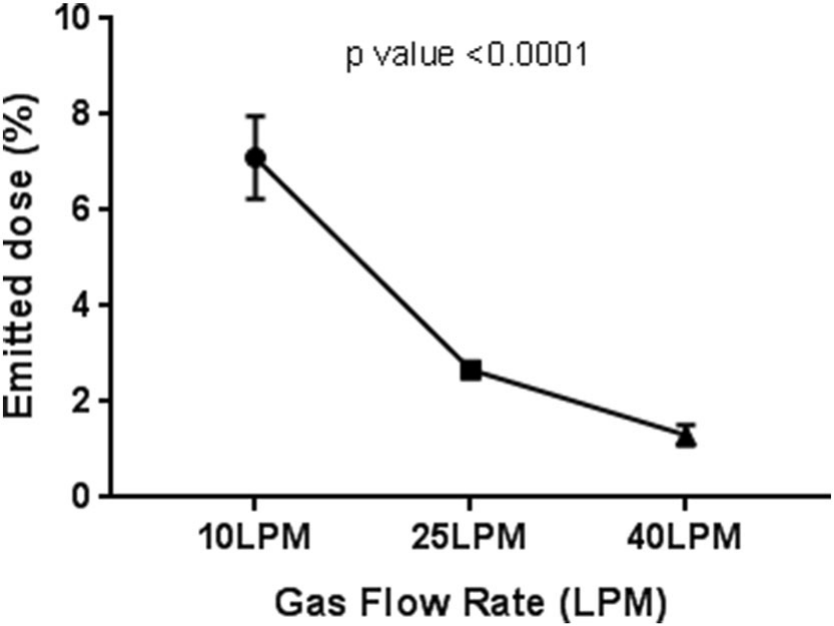
Effect of gas flow rate on emitted dose

Increasing the gas flow rate reduced the emitted dose, with a dose of 7.10% ± 0.86 obtained at 10 LPM, 2.67% ± 0.14 at 25 LPM, and 1.30% ± 0.21 at 40 LPM (Fig. 3). A one-way ANOVA was conducted to establish if the emitted dose varied statistically significantly with gas flow rate. The one-way ANOVA resulted in a *p* value of < 0.0001, indicating that different gas flow rates yielded significantly different emitted doses. A multiple comparison test (Tukey’s test) showed that gas flow rates of 10 and 25 LPM gave significantly different emitted doses (*p* < 0.0001), as did 10 and 40 LPM (*p* < 0.0001). The difference between the emitted doses obtained with gas flow rates of 25 and 40 LPM was not significant (*p* value = 0.0789).

### Influence of Input Droplet Size on Emitted Dose

**Fig. 4:**
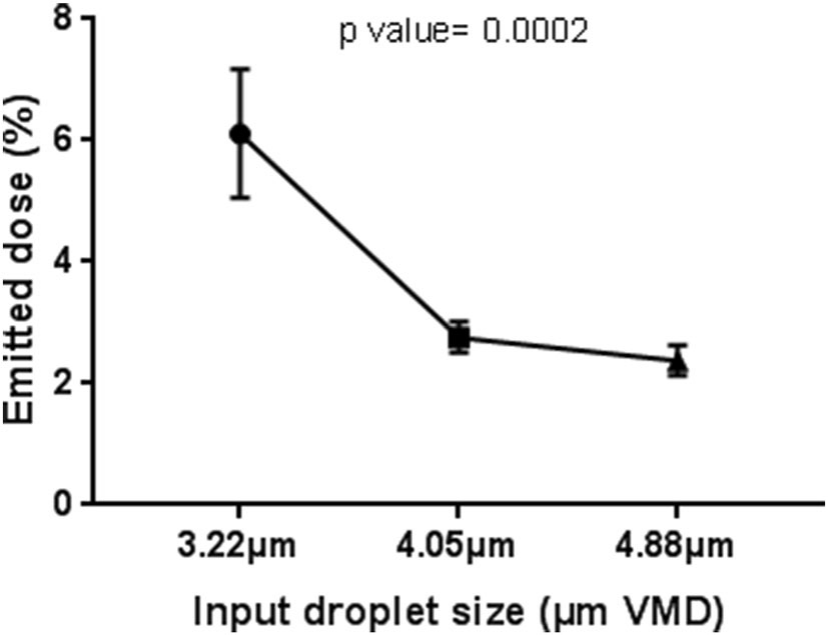
Effect of input droplet size on emitted dose

Increasing the input droplet size reduced the emitted dose, with a dose of 6.11% ± 1.06 obtained with a droplet size of 3.22 µm, 2.76% ± 0.25 with 4.05 µm, and 2.38% ± 0.25 with 4.88 µm (Fig. 4). A one-way ANOVA was conducted to establish if different droplet sizes yielded significantly different emitted dose between input droplet sizes (3.22, 4.05, and 4.88 µm). The one-way ANOVA resulted in a *p* value of 0.0002, indicating that nebulizers with different input droplet sizes yield significantly different emitted doses. This relationship generated a Pearson correlation coefficient (*r*) of − 0.2871. A multiple comparison test (Tukey’s test) between nebulizers with input droplet sizes of 3.22 and 4.05 µm provided significantly different emitted doses (*p* = 0.0008), as did the nebulizers with input droplet sizes of 3.22 and 4.88 µm (*p* = 0.0004). The difference in the emitted doses of nebulizers with input droplet sizes of 4.05 and 4.88 µm was not significant (*p* = 0.9029).

### Influence of Nebulizer Position on Emitted Dose

**Fig. 5:**
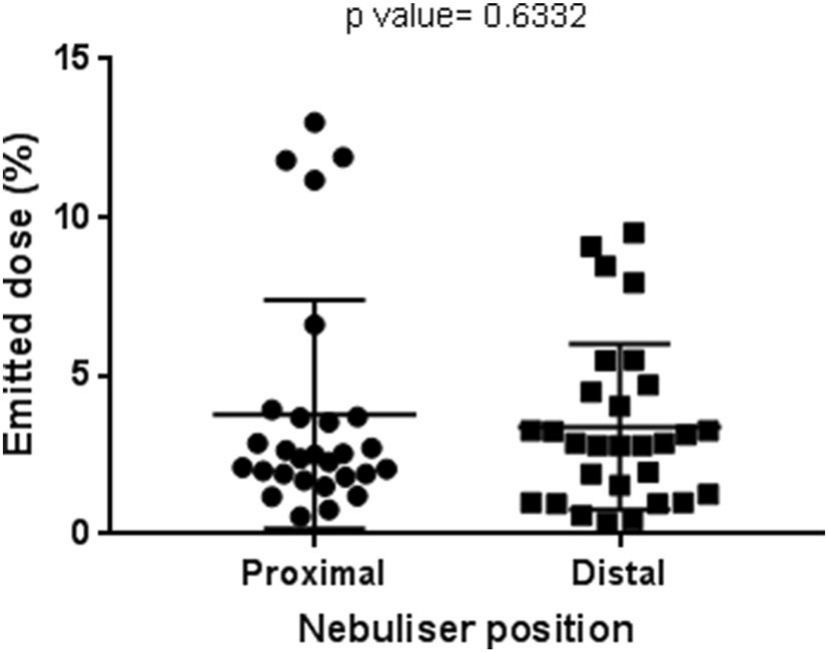
Effect of nebulizer position on emitted dose

Mean ± standard deviation values of the emitted dose were 3.79 ± 0.68%  for a proximal nebulizer placement position (immediately after the humidification chamber) and 3.39 ± 0.49% for a distal nebulizer placement position (immediately upstream of the nasal cannula) (Fig. 5). A Student’s *t* test was performed to determine if the emitted dose varied significantly depending on the nebulizer position in the humidification circuit. The Student’s *t* test resulted in a *p* value of 0.6332, indicating that switching the nebulizer position resulted in a small but not statistically significant change in emitted dose.

### Influence of Nasal Cannula Design on Emitted Dose

The doses emitted when using two adult nasal cannula interfaces (Comfort Flo and Comfort Flo Plus, Hudson RCI Neptune, Teleflex Medical) with the same HFNT circuit were compared. A nebulizer of midpoint droplet size (4.05 µm) was used. Testing was also completed at three gas flow rates (10, 25, and 40 LPM). The mean ± standard deviation values of emitted dose are presented in Table 8.

**Table 8:** Effect of nasal cannula design (delivery gas flow rate and cannula type) on emitted dose

Delivery gas flow (LPM)	Comfort Flo	Comfort Flo Plus
10	6.62	55.65 ± 1.19
25	2.62 ± 0.77	49.89 ± 1.93
40	0.77	40.40 ± 0.98

### Emitted Dose Using Pediatric and Infant Cannulas

The mean ± standard deviation values of emitted dose (%) from the pediatric and infant nasal cannulas are outlined in Tables 9 and 10, respectively. Just as seen when testing the adult cannula, a greater dose was emitted when a low gas flow rate was used with a small input droplet size and a proximal nebulizer position.

**Table 9:** Mean ± standard deviation values of emitted dose (%) for testing completed using a pediatric cannula (Comfort Flo P/N: 2411-02, Teleflex Medical)

Input droplet size (µm VMD)	Delivery gas flow rate (LPM)	Nebulizer position	Sample number (*n*)	Emitted dose (%)
3.22	5	Proximal	*n* = 3	18.73 ± 1.50
		Distal	*n* = 3	8.46 ± 0.64
	20	Proximal	*n* = 3	2.35 ± 0.35
		Distal	*n* = 3	1.21 ± 0.36
4.05	12.5	Proximal	*n* = 3	4.31 ± 0.79
		Distal	*n* = 3	2.02 ± 0.19
4.88	20	Proximal	*n* = 3	1.05 ± 0.26
		Distal	*n* = 3	1.07 ± 0.07

**Table 10:** Mean ± standard deviation values of emitted dose (%) for testing completed using an infant cannula (Comfort Flo P/N: 2411-03, Teleflex Medical)

Input droplet size (µm VMD)	Delivery gas flow rate (LPM)	Nebulizer position	Sample number (*n*)	Emitted dose (%)
3.22	2	Proximal	*n* = 3	18.03 ± 1.05
		Distal	*n* = 3	6.81 ± 0.78
	8	Proximal	*n* = 3	4.55 ± 0.61
		Distal	*n* = 3	1.36 ± 0.31
4.05	5	Proximal	*n* = 3	7.27 ± 1.12
		Distal	*n* = 3	1.86 ± 0.12
4.88	8	Proximal	*n* = 3	2.14 ± 0.38
		Distal	*n* = 3	1.10 ± 0.15

### Aerosol Droplet Size Measurement

The MMAD and GSD for each nebulizer under test was first characterized. Results from droplet size characterization at gas flow rates of 10 and 40 LPM are shown in Table 11. The droplet size exiting the nasal cannula interface was consistently lower than that produced by the nebulizer (input droplet size). In addition, the droplet size at the adult nasal cannula outlet decreased with increasing gas flow rate: 1.82 µm at 10 LPM and 1.72 µm at 40 LPM with an input droplet size of 3.22 µm VMD, 1.90 µm at 10 LPM and 1.72 µm at 40 LPM with an input droplet size of 4.05 µm VMD, and 1.68 µm at 10 LPM and 1.31 µm at 40 LPM with an input droplet size of 4.88 µm VMD.

**Table 11:** Mean ± standard deviation values of MMAD and GSD for each input droplet size at the nasal cannula with two different gas flow rates

Input droplet size			Adult cannula at 10 LPM		Adult cannula at 40 LPM	
VMD (µm)	MMAD (µm)	GSD (µm)	MMAD (µm)	GSD (µm)	MMAD (µm)	GSD (µm)
3.22	3.23 ± 0.04	2.36 ± 0.02	1.82 ± 0.18	1.45 ± 0.06	1.72 ± 0.08	1.64 ± 0.40
4.05	3.38 ± 0.27	2.49 ± 0.06	1.90 ± 0.09	1.46 ± 0.06	1.72 ± 0.14	3.22 ± 1.76
4.88	4.62 ± 0.11	2.31 ± 0.07	1.68 ± 0.09	1.72 ± 0.19	1.31 ± 0.10	1.73 ± 0.43

## Discussion

The use of concurrent aerosol delivery during HFNT may be employed to facilitate the delivery of a variety of prescribed medications for inhalation. This study details a systematic approach to establish the conditions required to optimize the emitted dose during HFNT. The well-established design of experiments approach provided a robust model, thus generating usable and representative data without committing to exhaustive testing. For the majority of the testing completed in the study, nasal cannula interfaces with small inner bore diameters were utilized. Consequently, emitted dose (%) results were relatively low. Nevertheless, the purpose of this study was not to yield high amounts of aerosol, but to determine the settings necessary to optimize the emitted dose, regardless of the circuit and nasal cannula interface used. Increasing the gas flow rate was found to significantly reduce the emitted dose, from 7.10% at 10 LPM to 2.67% at 25 LPM and 1.30% at 40 LPM (*p* < 0.0001). We found that a small input droplet size resulted in a significantly greater amount of aerosol exiting the cannula outlet, with a dose of 6.11% obtained with an input droplet size of 3.22 µm, 2.76% with 4.05 µm droplets, and 2.38% with 4.88 µm droplets (*p* = 0.0002). In addition, the droplet size exiting the nasal cannula interface was smaller than that produced by the aerosol generator for all devices under test. Positioning the nebulizer immediately after the humidification chamber yielded a marginally greater emitted dose (3.79%) than when the nebulizer was placed immediately upstream of the nasal cannula (3.39%). Flow rate, input droplet size, and nebulizer position were at the 0.10 level of significance, indicating that all three factors had a significant effect on emitted dose. Overall, flow rate had the greatest effect on emitted dose, followed by input droplet size and then nebulizer position (Fig. 2).

### Influence of Flow Rate on Emitted Dose

According to the statistical DOE model, flow rate was the factor that had the greatest effect on emitted dose. Increasing the gas flow rate significantly reduced the dose emitted with an adult nasal cannula, from 7.10% ± 0.86 at 10 LPM to 2.67% ± 0.14 at 25 LPM and 1.30% ± 0.21 at 40 LPM (*p* < 0.0001) (Fig. 3). Similarly, the emitted dose was flow-rate dependent when utilizing pediatric and infant nasal cannulas (Tables 9, 10). This is consistent with the findings of existing studies in the literature. Higher gas flow rates most likely caused greater depositional losses within the circuit tubing in comparison to when testing with lower flow rates. Ari et al. reported an aerosol delivery efficiency of ~ 10% at a flow rate of 3 LPM, and this decreased significantly to ~ 2% as the flow rate was increased to 6 LPM [13]. Similarly, Perry et al. reported that the in vitro inspired dose during HFNT decreased from 2.5% to 0.2% upon increasing the flow rate from 5 to 40 LPM [15]. Reminiac et al. also showed reductions in inhaled dose with increasing gas flow rate [22].

### Influence of Input Droplet Size on Emitted Dose

Our findings indicate that there was a significant difference in the doses emitted by nebulizers with different input droplet sizes, with an increasing input droplet size associated with a reduced emitted dose (6.11% ± 1.06 at 3.22 µm, 2.76% ± 0.25 at 4.05 µm, and 2.38% ± 0.25 at 4.88 µm) (Fig. 4), with a Pearson correlation coefficient (*r*) of − 0.2871. One-way ANOVA resulted in a *p* value of 0.0002, indicating that there was a significant difference in emitted dose between nebulizers with different input droplet sizes. A similar trend was observed when testing with pediatric and infant nasal cannulas (Tables 9, 10).

Furthermore, the droplet size was subsequently characterized at the cannula outlet at gas flow rates of 10 and 40 LPM. MMAD is the diameter that is larger than 50% of the droplets and smaller than the other 50%, based on mass, and it is considered the best predictor of aerosol distribution throughout the lungs. Droplets less than 5 µm in size are predicted to deposit in the lungs of adults, whilst larger droplets are more likely to deposit in the upper airways [23, 24]. The droplet size exiting the nasal cannula interface was consistently lower than that produced by the nebulizer (input droplet size) for all three devices under test (Table 11). This finding is consistent with that of Bhashyam et al., who noted a similar decrease in aerosol size measurements at various points in a HFNT system [12]. This reduction is likely caused by inertial impaction within the delivery system, enabling aerosol droplets of a smaller droplet size to successfully transit through [12]. Those results suggest that losses throughout the circuit may be reduced by applying aerosol generators that produce a low MMAD initially [21, 25, 26].

We found that droplet size at the adult nasal cannula outlet decreased with increasing gas flow rate (10–40 LPM), in contrast with some studies in the literature. Reminiac et al. reported that larger particle size measurements were associated with high flow rates [21]. These contrasting results may be explained by the use of different test setups. Reminiac and colleagues placed the nasal cannula at the entry to the throat of the cascade impactor, whereas the nasal prongs were presented to the throat of the NGI via a fixture in this study. This fixture was employed to minimize rained-out humidity that may artificially increase droplet size measurements.

### Influence of Nebulizer Position on Emitted Dose

The position of the nebulizer during simulated mechanical ventilation has previously been shown to exert a critical influence on the dose delivered, due to the aerosol being exposed to bias flow [27, 28]. The effect of nebulizer position during HFNT is not, however, well described. In this study, positioning the nebulizer immediately after the humidification chamber led to a larger emitted dose (3.79 ± 0.68%) than when the nebulizer was placed immediately upstream of the nasal cannula (3.39 ± 0.49%), although the difference in emitted dose was not statistically significant (Fig. 5). Placing the nebulizer close to the humidification chamber yielded a marginally higher emitted dose than the doses emitted when the nebulizer was placed in distal positions, such as immediately upstream of the nasal cannula, due to the point of entry of the aerosol into humidified/nonhumidified air [13, 15]. Reminiac et al. studied the effect of nebulizer position on aerosol delivery during simulated HFNT for adults in vitro. Nebulizers were attached before the humidification chamber, after the humidification chamber, or at the distal position immediately upstream from the nasal cannula. Reminiac and colleagues showed that when using the Aerogen-mesh nebulizer, the inhalable mass was significantly lower at the distal position immediately upstream of the cannula [22].

### Influence of Nasal Cannula Design on Emitted Dose

For the majority of the testing, nasal cannula interfaces with small dimensions were utilized. Consequently, emitted dose results were quite low. Nevertheless, the purpose of this study was not to yield high amounts of aerosol from nasal cannula, but to identify the conditions required to optimize the emitted dose, regardless of the circuit and interface used. A greater emitted dose was observed when the Comfort Flo Plus cannula was used rather than the Comfort Flo cannula. This is most likely explained by the larger inner bore diameter of the Comfort Flo Plus nasal cannula interface. Small dimensions in the tubing and cannula components hinder the exit of aerosol from the cannula during HFNT [29]. Perry et al. reported a decrease in inspired dose during HFNT when smaller cannulas were utilized (pediatric and infant) rather than adult cannulas [15]. Pacocha et al. showed that the brand of nasal cannula interface also affects aerosol delivery in HFNT during simulated normal adult breathing [30].

### Clinical Implications

Several factors influence both the amount of aerosol exiting the cannula and the amount available to the patient during HFNT. The factors affecting the emitted dose have been systemically reviewed in this study and our findings may be clinically relevant as they could help to optimize the delivery of a variety of prescribed medications for inhalation. These include, but are not limited to, β-agonists and hypertonic saline [4, 5]. However, efficient aerosol delivery to the lungs during HFNT is challenging due to the high gas flow rates used clinically and the aforementioned small dimensions of the delivery tubing and nasal cannula. Furthermore, the nasal passages present a challenge for the aerosols to navigate before reaching the lungs [29]. The first in vivo study on aerosol delivery during HFNT reported low pulmonary aerosol deposition in healthy male subjects (1–4% of the dose placed in the nebulizer), regardless of the type of nebulizer used [31]. Therefore, the refinement of aerosol delivery systems may be necessary. A number of studies have assessed the effect of breathing pattern on aerosol delivery during HFNT and have obtained contrasting results. Sweeney et al. showed that the mean tracheal dose during simulated HFNT in vitro is significantly lower for a cystic fibrosis breathing profile than for normal adult breathing [32]. Conversely, Reminiac et al. reported that the respirable mass of drug was significantly higher during simulated respiratory distress than simulated quiet breathing [22]. Dailey et al. showed that with a distressed breathing pattern, aerosol delivery was greater at 30 and 50 LPM than with a quiet breathing pattern [33].

### Study Limitations

The intention of this study was to identify the main effects contributed by a high-flow therapy system design. This study did not assess how aerosol efficiency during HFNT is affected by simulated breathing, using an anatomically correct model. Future studies are required to investigate how patient factors affect aerosol delivery during HFNT, including breathing patterns and patient size (adult, pediatric, and infant).

## Conclusion

In conclusion, our findings indicate that to optimize the amount of aerosol exiting the nasal cannula during HFNT, the gas flow rate should be low and the input aerosol droplet size should be small, while the nebulizer should be positioned close to the humidifier. Gas flow rate was the factor that had the greatest influence on emitted dose, followed by input droplet size and then nebulizer position. Whilst only a single commercially available high-flow therapy circuit was used in this assessment, clinicians, users, and system designers may nevertheless benefit from this comprehensive analysis of relevant factors when they attempt to optimize the delivery of medications for inhalation. Future studies may further investigate the factors that affect the amount of aerosol available to the patient during HFNT, including breathing patterns and patient size.
